# Evolution in Pathology

**Published:** 1887-04

**Authors:** 


ARTICLE VIII.

### EVOLUTION IN PATHOLOGY.

### MAN'S LOST INCISORS.

In his lectures on "Evolution in Pathology," delivered at the Royal College of Surgeons of England, Mr. J. Bland Sutton, F. R. C. S., referring to the suppression of parts, said that one of the clearest instances of suppression, and at the same time one capable of indisputable demonstration, is connected with the disappearance and occasional reappearance of a third incisor tooth in man. The matter was first worked out and announced by Professor Albrecht (now of Hamburg) in quite a number of papers, that normally man inherits three incisors on each side in the upper maxillæ, but during development the middle (second) one of the three is suppressed. In many cases of cleft palate, however, more room is afforded, and the usually suppressed tooth attains a functional condition. The question was one of importance, and I was able in a paper read before the Odontological Society of Great Britain in December, 1884, to confirm this part of Albrecht's observation. Professor Sir W. Turner, a month later, adduced also confirmatory testimony in the *Journal of Anatomy and Physiology*, and the last contribution in this direction is by Windle and Humphrey in the same journal. A careful analysis of the facts shows, beyond all doubt, that in the usual course of events an incisor tooth is suppressed in the upper maxilla of man; the only point admitting of any latitude of opinion is whether the missing tooth is the second or third incisor. As the case stands at present the balance of opinion is in favour of it being the second.

We must not forget, however, that supernumerary teeth are found in other situations than in the incisor series; indeed, they may occur in almost any part of the dental arch, and may vary in character from a perfectly formed enamel-covered tooth to a tiny conical mass of dentine. In determining whether an extra tooth is a supernumerary one or not, we must also take into consideration the fact that an excess in the number of teeth is occasionally due to the retention of one or more milk teeth. In order to comprehend the true significance of supernumerary teeth, it is necessary to bear in mind the morphology of these organs. In their essential features the teeth of a shark agree with those of a mammal, and in their development as calcified papillæ of the involuted epiblast in the buccal region—the stomodæum—the two forms are in perfect harmony. In the case of the shark almost the whole of the mouth is beset with teeth, whereas in mammals they are normally restricted to certain very definite tracts. An unprejudiced survey of the facts ought to convince us that though the teeth of mammals are thus kept within narrow limits, yet the papillæ in the immediate vicinity of these teeth territories are potentially teeth, and it is perfectly consonant with what we know of the principles of atavism that these papillæ should occasionally declare their ancestry by developing as rudimentary, or even perfect, teeth. Nor is this form of atavism limited to this particular region; for, inasmuch as teeth are modified papillæ of the skin or integumental covering (and this may be absolutely demonstrated in the case of the young of the dog-fish, in whom the various stages may be clearly traced from placoid scales to teeth), so in those remarkable teratomata arising in obsolete canals lined with epiblastic tissues, calcified papillæ (teeth) make their appearance. Dentine and enamel are tissues which exist in scanty proportions in man, yet they formerly occurred in great abundance in the remarkable mailed-ganoids which are encased in an elaborate armour of these very extraordinary tissues.

If we admit the above opinions, then a rational explanation is forthcoming of certain interesting pathological conditions which occur in the mouth. For instance, some form of odontomata may be considered as aberrant involutions of buccal epiblast and papillæ; the view is supported by the fact that this variety of neoplasm occurs in many mammals. There is also good evidence to support the view that the milk dentition is to be regarded as a set of teeth appearing in obedience to the law of inheritance. In many mammals they are, like the lanugo of the human fœtus, shed before the embryo quits the uterus. If supernumerary teeth can be regarded as atavistic, then we must consider certain pre-calcific stages of teeth in the same light; for instance, in the early stage a tooth consists of an up-growing papilla capped by a down-growth of epithelium. Suppose the development to advance no further, but growth to continue, the result is an aberrant formation—a neoplasm. Involutions of this kind have been detected by Malassez, and Mr. Eve has discussed their relation in connection with multilocular cystic tumours of the jaws. The upshot of the argument is this: supernumerary teeth, odontomata (excluding the cementomata of herbivora), and multilocular cystic tumours of the jaws may be regarded as originating in the germs of teeth suppressed in the process of evolution of our species The incisor tooth, to the consideration of which the early part of the argument was dedicated, may be considered, in all probability, as the last tooth in the order of suppression.

In addition to neoplasms originating in undeveloped enamel germs, we have to take into consideration a class of tumour usually described somewhat vaguely as adenomata, growing from the palate. Our knowledge of these cases is much advanced by the work of Mr. Stephen Paget on this subject. It appears that neoplasms, perfectly innocent in their nature, but full of epithelial nests, occur in the palate, and there are good grounds for believing that many of the growths variously described as glandular, alveolar sarcoma, alveolar carcinoma (!), &c., have their origin in little round masses of epiblast, which become enclosed between the two horizontal plates, which fuse together in the median line in order to separate the nasal and buccal cavities. The existence of such isolated epithelial islets has been affirmed by more than one observer, and a good account of them, with references to the literature of the subject, will be found in Leboucq's papers on "Le Canal Naso-palatine chez l'Homme," and "Note sur les Perles Epitheliales de la Voute Palatine."—*The London Dental Record.*

---

